# The Estimated Electrode-Neuron Interface in Cochlear Implant Listeners Is Different for Early-Implanted Children and Late-Implanted Adults

**DOI:** 10.1007/s10162-019-00716-4

**Published:** 2019-03-25

**Authors:** Mishaela DiNino, Gabrielle O’Brien, Steven M. Bierer, Kelly N. Jahn, Julie G. Arenberg

**Affiliations:** 1 Department of Psychology, Carnegie Mellon University, 5000 Forbes, Ave., Pittsburgh, PA 15213 USA; 2 Department of Speech and Hearing Sciences, University of Washington, 1417 NE 42nd St., Box 354875, Seattle, WA 98105 USA; 3 Department of Otolaryngology, Massachusetts Eye and Ear, Harvard Medical School, 243 Charles St., Boston, MA 02114 USA

**Keywords:** cochlear implant, children, threshold, dynamic range, impedance, electrical field imaging

## Abstract

Cochlear implant (CI) programming is similar for all CI users despite limited understanding of the electrode-neuron interface (ENI). The ENI refers to the ability of each CI electrode to effectively stimulate target auditory neurons and is influenced by electrode position, neural health, cochlear geometry, and bone and tissue growth in the cochlea. Hearing history likely affects these variables, suggesting that the efficacy of each channel of stimulation differs between children who were implanted at young ages and adults who lost hearing and received a CI later in life. This study examined whether ENI quality differed between early-implanted children and late-implanted adults. Auditory detection thresholds and most comfortable levels (MCLs) were obtained with monopolar and focused electrode configurations. Channel-to-channel variability and dynamic range were calculated for both types of stimulation. Electrical field imaging data were also acquired to estimate levels of intracochlear resistance. Children exhibited lower average auditory perception thresholds and MCLs compared with adults, particularly with focused stimulation. However, neither dynamic range nor channel-to-channel threshold variability differed between groups, suggesting that children’s range of perceptible current was shifted downward. Children also demonstrated increased intracochlear resistance levels relative to the adult group, possibly reflecting greater ossification or tissue growth after CI surgery. These results illustrate physical and perceptual differences related to the ENI of early-implanted children compared with late-implanted adults. Evidence from this study demonstrates a need for further investigation of the ENI in CI users with varying hearing histories.

## Introduction

Newborn hearing screening has led to earlier detection of hearing impairments, allowing more children with severe-to-profound hearing loss to receive cochlear implants (CIs) at young ages (e.g., Boons et al. 2013; Halpin et al. 2010; Lammers et al. 2015). In contrast, many current adult CI users maintained acoustic hearing until later in life and were implanted at older ages. These divergent demographics may result in differences in the interface between CI electrodes and spiral ganglion neurons between these two populations (Bierer 2010).

The electrode-neuron interface (ENI) refers to the effectiveness with which each electrode stimulates its target spiral ganglion neurons. Suboptimal interfaces can result from electrodes positioned distant from target neurons (Finley et al. 2008), degeneration of those neurons (Miura et al. 2002), or bone and tissue growth near stimulating electrodes (Seyyedi and Nadol 2014). Systematic differences in the number of poorly positioned electrodes between children and adults with CIs are unlikely (e.g., Noble et al. 2014, 2016). However, age at implantation, duration of deafness, and etiology of deafness differ substantially between these groups, likely influencing neural health. Degeneration of peripheral and central auditory system neurons occurs in the absence of auditory input (Otte et al. 1978) and with normal aging (Makary et al. 2011). Early-implanted children may have healthier auditory neurons compared with adults who received a CI years after diagnosis of severe-to-profound hearing loss.

To assess the quality of the ENI in adult CI users, several investigations have narrowed the spatial extent of electrical current spread by routing the return current path primarily within the cochlea (Bierer 2007; Kral et al. 1998; Snyder et al. 2004). This focused stimulation is believed to be more sensitive to ENI quality than monopolar stimulation: several studies in adults have found relatively uniform monopolar auditory perception thresholds across the CI array within individual subjects, but highly variable focused thresholds for the same individuals (e.g., Bierer 2007). Variability in channel-to-channel thresholds likely reflects variation in the quality of the ENI, which may contribute to inconsistent transmission of spectral information. Accordingly, prior investigation in adults with CIs showed that greater focused threshold variability was associated with lower speech recognition scores (Long et al. 2014). However, little is known about auditory perceptual thresholds across the electrode array in children. One goal of the current study was to utilize thresholds to assess the quality of the ENI of early-implanted pediatric CI users.

In adults with CIs, channels with relatively high focused thresholds often have small dynamic ranges, defined as the difference between threshold and most comfortable level (MCL; Bierer and Nye 2014). Relatively low MCLs may also reduce dynamic range for channels with suboptimal ENIs. Small dynamic ranges in adults (Firszt et al. 2002) and children (Robinson et al. 2012) correlate with poorer speech identification performance, which could be particularly detrimental to children who are still developing spoken language. Yet, the relation between assessments of the ENI and dynamic range in children was unknown.

Electrical field imaging (EFI) is another technique that could provide information about the effect of hearing demographics on the cochlear environment. EFI uses the built-in telemetry systems of modern CIs to measure how intracochlear potential is distributed in the cochlea (for review, see Mens 2007). Increases in impedance of the CI electrode contacts have been related to bone and tissue growth around the electrode array in adults (Wilk et al. 2016). Prior studies have observed higher electrode impedance levels in pediatric relative to adult CI users (Busby et al. 2002; Hughes et al. 2001; Molisz et al. 2015), suggesting more extensive cochlear ossification and tissue growth in children. EFI evaluates electrode impedance as well as that of the surrounding cochlear tissue and could thus provide information that is not captured by clinical impedance measures (Vanpoucke et al. 2004). This technique may provide evidence of anatomical and physiological differences between children and adults that are pertinent to clinical practice.

At present, investigations of the ENI using focused thresholds and EFI measures have been conducted primarily in late-implanted adults (e.g., Bierer 2007; Finley et al. 2008; Long et al. 2014). However, early-implanted children and late-implanted adults have distinct demographics that likely influence ENI quality. This study tested the hypothesis that factors related to the ENI would differ between these groups. Further, investigation of the ENI in early-implanted children could provide insight into the effects of electrical stimulation after early hearing loss.

## Methods

Eleven children aged 11 to 17 (mean age = 14.06, standard deviation = 1.9 years) and eleven adults aged 48 to 84 (mean age = 63.08, standard deviation = 11.6 years) with CIs participated in this study. Half of the children (P02, P03, P10, P11, and P12) failed a newborn hearing screening. The other children were diagnosed with severe-to-profound hearing loss prior to age four (P04, P05, P06, P07, P08, and P09). All children received their first implant before age 5 (mean age at implantation = 2.17, standard deviation = 1.2 years). All adults were born with acoustic hearing, were diagnosed with severe-to-profound hearing loss in adulthood (mean age at diagnosis = 32.6, standard deviation = 13.2 years), and received their first CI at much older ages compared with the children (mean age at implantation = 56.1, standard deviation = 13.5 years). Ten children and three adults were bilaterally implanted; however, due to the large difference in number of bilaterally implanted individuals between the groups, only first-implanted CIs were included in this study. Participant demographics and speech identification scores are in Table 1. Speech identification performance was assessed with medial vowels presented in /hVd/ context. Stimuli were played through speakers in a sound-attenuating booth. For bilateral and bimodal listeners, the contralateral CI or hearing aid was turned off during this test (for detailed methods, see DeVries et al. 2016; DiNino and Arenberg 2018).

**Table 1:** Demographic information

Subject:	Gender:	Etiology of deafness:	Ear:	Age at testing:	Age at implantation (years):	Duration of deafness (years):	Vowel identification score:
Children							
P02	M	EVA	R	11.8	1.1	1.0	87.0%
P03	M	Unknown	R	12.9	1.4	1.1	95.0%
P04	F	Unknown	R	13.2	1.5	0.8	77.0%
P05	M	DFNB1	R	17.7	4.1	3.0	96.5%
P06	F	Unknown	R	17.2	4.3	2.5	100 %
P07	F	Unknown	R	13.3	1.9	0.4	100 %
P08	M	EVA	L	15.3	2.9	0.7	68.5%
P09	F	Unknown	L	13.5	2.6	1.3	82.0%
P10	M	DFNB1	L	13.3	1.1	0.9	93.0%
P11	F	DFNB1	R	13.3	1.4	1.2	52.0%
P12	M	DFNB1	R	13.3	1.7	1.4	62.7%
Mean				14.1	2.2	1.3	83.1%
SD				1.9	1.2	0.8	16.3%
Adults							
S28	F	Autoimmune	R	74.9	69.7	18.8	51.5%
S29	M	Unknown	L	84.0	76.8	30.3	93.0%
S38	M	Otosclerosis	L	49.8	46.2	28.3	41.3%
S39	F	Hereditary	R	53.4	30.1	9.1	100 %
S41	M	Maternal Rubella	L	48.8	42.9	1.2	100 %
S45	F	Hereditary	R	62.7	54.0	32.0	100 %
S46	M	Unknown	R	67.2	64.2	48.2	52.3%
S48	F	Autoimmune	R	59.4	58.0	22.0	86.5%
S50	F	Measles	R	76.5	61.1	41.1	53.3%
S52	F	Unknown	R	70.1	66.0	21.1	82 %
S55	F	Hereditary	R	63.7	48.5	7.3	100 %
Mean				64.6	56.1	23.6	78.2%
SD				11.4	13.5	14.3	23.6%

Demographic information for all child and adult participants, including gender, etiology of deafness, age on testing date, age at implantation, duration between diagnosis of severe-to-profound hearing loss and receipt of a CI, and medial vowel identification scores in quiet with that CI

EVA, enlarged vestibular aqueduct; DFNB1, genetic nonsyndromic hearing loss

Pediatric and adult CI users were recruited from hospitals and audiology clinics in the greater Seattle area. All participants used oral communication and were native speakers of American English. To minimize potential sources of variability, all participants utilized Advanced Bionics HiRes 90K devices and HiFocus 1J electrode arrays. In addition, individuals with electrode positioners were excluded from this study because (1) positioners are designed to place the electrodes closer to target neurons, and distance could affect auditory detection thresholds (e.g., DeVries et al. 2016; Long et al. 2014), and (2) intracochlear impedance as assessed by EFI are likely sensitive to the volume of the fluid-filled space around the electrode array; positioners fill much of that space and could alter the impedance estimates.

### Assessments

#### Auditory Detection Thresholds

Thresholds with monopolar and focused electrical stimulation were obtained using a fast sweep procedure with current steering (as in Bierer et al. 2015a, and based on Sek et al. 2005). Focused stimulation was implemented with the steered quadrupolar (sQP) electrode configuration, which involves four intracochlear electrodes: the two middle electrodes serve as active electrodes and the two outer electrodes serve as the returns. Sigma (σ) specifies the amount of current delivered through the intracochlear return electrodes, with the remainder flowing through an extracochlear ground. Greater sigma values indicate greater current focusing, such that σ = 0 represents monopolar stimulation, in which all return current flows through the extracochlear electrode, and σ = 1 represents the greatest degree of focusing, in which the intracochlear electrodes carry all return current.

The sQP configuration allows current steering between the two active electrodes. Alpha (ɑ) is the steering coefficient: when ɑ = 0, all steered current passes through the more apical active electrode; when ɑ = 1, all steered current passes through the more basal active electrode. Electrode channel number (i.e., “channel 4”) is typically defined by the basal active electrode. However, because the sQP configuration requires four electrodes, this arrangement is not possible to steer current for electrode 2. Instead, the same set of electrodes as steering for channel 3 are used, and an α value of 0 is set to center the current on electrode 2. This arrangement is “channel 2” despite electrode 2’s position as the apical active electrode.

Thresholds were obtained with σ = 0 (monopolar) and σ = 0.9 (focused) electrode configurations. The sigma value for focused thresholds was chosen because it allowed for the presentation of current levels that were perceptible but below voltage compliance limits. These limits, defined as the maximum voltage supported by the device divided by the impedance of that electrode channel, were calculated for each subject at the beginning of the test session. For one participant (P08), however, a sigma value of 0.8 was used because this subject’s thresholds with σ = 0.9 could not be obtained without reaching voltage compliance limits. As the sigma value affects threshold levels, sensitivity analyses were performed by removing this subject’s data. The overall findings of this study were unaffected.

Stimuli were biphasic, charge-balanced pulse trains with the cathodic phase leading. Pulses were 97 microseconds (μs) in duration and were presented at a rate of 997.9 pulses per second. Each pulse train was 200.4 milliseconds (ms) long. The Bionic Ear Data Collection System (BEDCS version 1.18.315; Advanced Bionics, LLC) and custom software in MATLAB (The MathWorks, Inc.) were used to present stimuli and record participant responses.

MCL was first determined behaviorally for each channel using the Advanced Bionics clinical loudness scale (Advanced Bionics, LLC). This scale ranges from “1” (“Just Noticeable”) to “10” (“Too Loud”). Current level was increased manually until subjects reported a loudness rating of “6” (“Most Comfortable,” a loudness level that is comfortable to listen with for an extended period of time). MCL was then set as the upper limit for stimulation, ensuring that presented current levels for each participant would not exceed comfortable levels.

For each electrode configuration, participants performed threshold sweeps of electrodes 2 through 15 in which ɑ was changed in steps of 0.1. Thresholds for electrodes 1 and 16 were not acquired because the focused electrode configuration necessitates flanking electrodes. Participants performed one forward sweep (from apical to basal electrodes) and one backward sweep (from basal to apical electrodes). Each sweep utilized a Békésy-like tracking procedure (Békésy 1947): the listener was instructed to hold down the spacebar when they could perceive the signal and to release the spacebar when they could no longer perceive it. Each electrode and alpha step is called a “channel.” For example, electrode 6 with ɑ = 0.4 is channel 6.4. The detection thresholds from the forward and backward sweep(s) utilized in this study were those averaged for each non-steered integer electrode channel; for example, channel 6.0.

Current level requirements varied greatly between the electrode configurations as well as subjects’ individual electrode channels, and thus the logarithmic decibel scale was used. This scale is considered to be more suitable than a linear scale for reporting such values because CI loudness growth functions are typically nonlinear (e.g., Chatterjee et al. 2000; Sanpetrino and Smith 2006). A one-way multivariate analysis of covariance (MANCOVA) was conducted to compare threshold profiles of early-implanted children and late-implanted adults. Focused and monopolar thresholds across the electrode array were entered as dependent variables and “Group” (child or adult) as the independent variable. “Electrode” was included as a covariate to control for site-specific effects in threshold profiles. Correction for multiple comparisons (for the two ANOVAs within the analysis) was performed using a Bonferroni adjustment (ɑ = 0.025).

#### Channel-to-Channel Variability

MATLAB (The MathWorks, Inc.) was utilized to assess the variation in monopolar and focused thresholds across the electrode array. The standard deviation of the signed differences in thresholds between each adjacent electrode was calculated from the threshold profiles of each participant. Use of the standard deviation instead of the mean difference between channels provides a measurement of the local variability, as opposed to the absolute magnitude of variability of all channels (e.g., Pfingst and Xu 2005). Calculations were performed separately for thresholds from each electrode configuration. A one-way multivariate analysis of variance (MANOVA) was performed to compare threshold variability values between children and adults. Channel-to-channel variability of focused and monopolar thresholds were dependent variables, with “Group” as the independent variable. A Bonferroni correction for multiple comparisons was performed at ɑ = 0.025.

#### Dynamic Range

MCL was chosen to represent the upper limit of the dynamic range instead of higher loudness ratings, such as upper limit of comfort level (ULCLs) or maximum acceptable levels (MALs), so that children would not be exposed to sounds that were louder than “comfortable.” The differences between MCL and threshold in dB for each electrode were calculated for each participant for both the monopolar and the focused electrode configurations. For a subset of channels for one child and for several adult subjects (P08, S29, S38, S41, S45, S50, S55), voltage compliance limits of the device were reached at current levels below MCL with focused stimulation. The level of current at compliance limit was not the true MCL for such channels, and therefore focused MCL and dynamic range values for those channels were not included in the analyses.

A one-way MANCOVA was conducted to compare dynamic range between early-implanted children and late-implanted adults. Monopolar and focused dynamic range were dependent variables, “Group” was the independent variable of interest, and “Electrode” was included as a covariate to account for variance in dynamic range due to electrode site.

The level of current used to define the upper limit of the dynamic range, the MCL, is also a measure related to the ENI and could potentially differ between early-implanted children and late-implanted adults. Therefore, an additional one-way MANCOVA testing a separate hypothesis was performed with focused and monopolar MCLs as the dependent variables. “Group” was set as the independent variable and “Electrode” was again included as a covariate to control for site-specific effects on MCL. A Bonferroni correction for multiple comparisons for each analysis was performed at ɑ = 0.025.

#### Intracochlear Impedance

Electrical field imaging (EFI) data were collected using BEDCS (version 1.18315; Advanced Bionics, LLC). The stimuli were biphasic monopolar pulses (100 μs in duration, 50 or 100 μA in amplitude) delivered at a rate of 16.6/s. Ten pulses were presented consecutively on each electrode site, while the voltage on every electrode was recorded sequentially at a 56 kHz sampling rate.

The data were analyzed offline using MATLAB (The MathWorks, Inc). After averaging, signal amplitude at each recording electrode was calculated as half the difference between the positive and negative voltage excursions, then scaled to units of resistance. Following the approach of Vanpoucke et al. (2004), the 16 × 16 matrix of EFI impedances was transformed to solve a lumped parameter resistor network representing current flow along and out of the cochlea. Three types of resistance were of interest: longitudinal (R_long_; resistance to current flow from the electrode along the length of the cochlea), transverse (R_trans_; resistance to current flow out of the cochlea to the ground electrode), and total (R_total_; resistance from all current pathways out of the cochlea) resistances. The resistor components of the network were labeled R_long_ and R_trans_ for each electrode position. The values were estimated using least squares optimization, with a localized weighting scheme to improve fitting of the EFI profiles and a regularization constraint to impose some degree of smoothness on the R_long_ and R_trans_ values across electrodes. One additional resistor value was defined for each stimulating electrode, as the peak of the reconstructed EFI profile (based on the solution to the ladder network). This value was termed R_total_, as it represented the total resistance encountered by the stimulation electrode, incorporating all possible current pathways in the resistor network model.

This analysis resulted in estimates for R_long_, R_trans_, and R_total_ for each electrode of a subject’s array. A one-way MANCOVA was performed to examine potential differences in these intracochlear resistance values between children and adults. R_trans_, R_long_, and R_total_ were dependent variables and “Group” was the independent variable. “Electrode” was included as a covariate to control for site-specific effects on intracochlear resistance. A Bonferroni correction for multiple comparisons was performed at ɑ = 0.017.

Clinical electrode impedance measurements for each electrode in each participant’s array were collected via SoundWave software (Advanced Bionics, LLC) at the beginning of the testing session in which EFI data were collected. A one-way univariate analysis of covariance (ANCOVA) was conducted to determine whether electrode impedance differed significantly between early-implanted children and late-implanted adults. “Electrode” was included as a covariate to account for any site-specific changes in electrode impedance.

A mixed-model regression analysis was performed to examine the relation between EFI impedance values and those of clinical electrode impedance. R_long_ was set as the independent variable in this model. This EFI component assesses resistance to current within the cochlea instead of resistance to current flowing out of the cochlea (e.g., Vanpoucke et al. 2004), and would thus be most affected by intracochlear tissue growth. Electrode impedance was the dependent variable and “Subject” was included as a random intercept to account for clustering of multiple electrode-specific measurements within each participant.

## Results

**FIG. 1:**
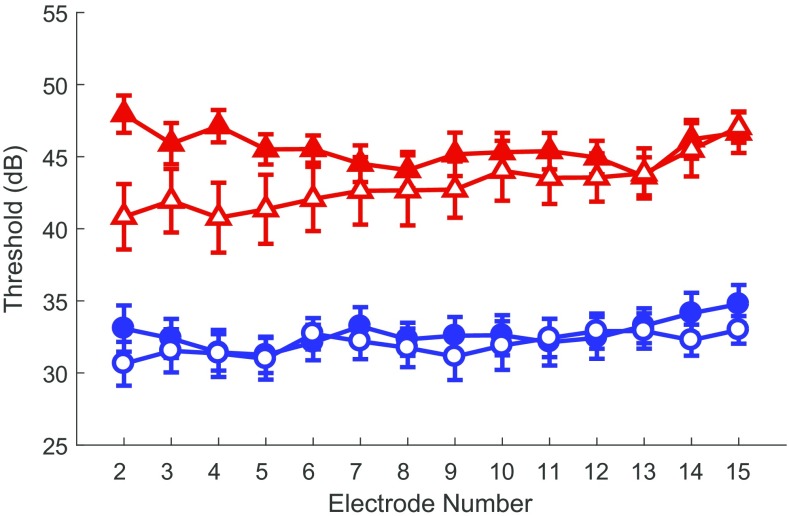
Auditory detection thresholds for late-implanted adults and early-implanted children across the electrode array. Blue circles indicate thresholds from the monopolar electrode configuration and red triangles indicate thresholds from the focused electrode configuration. Adult data are filled symbols and child data are open symbols. Error bars represent one standard error above and below the mean

Focused thresholds across the electrode array were higher than monopolar thresholds in early-implanted children as well as in late-implanted adults, consistent with previous studies conducted in adults with CIs (e.g., Bierer 2007). However, on average, children’s thresholds were lower than those of adults for both electrode configurations (see Fig. 1). Differences of 0.7 dB for monopolar thresholds and 2.6 dB for focused thresholds were observed between groups. Uncorrected group means and standard deviations for focused and monopolar thresholds are shown in Table 2. Statistical comparison of auditory detection thresholds between early-implanted children and late-implanted adults revealed a significant effect of group on thresholds (*F*(2,300) = 9.5, *p* < 0.001, Wilk’s ƛ = 0.941, partial *η*^2^ = 0.059) when controlling for electrode site. Focused thresholds were significantly different between the children and adults (*F*(1,301) = 16.1, *p* < 0.001, partial *η*^2^ = 0.051). Significant differences were not observed between groups for monopolar thresholds (*F*(1,301) = 1.9, *p* = 0.17, partial *η*^2^ = 0.006). No significant effects of electrode site on threshold profiles were found (*F*(2,300) = 2.5, *p* = 0.08, Wilk’s ƛ = 0.983) suggesting that group means were not significantly driven by thresholds from specific electrodes.

**Table 2:** Means and Standard Deviations of ENI Assessments

	Group			
	Children (*n* = 11)		Adults (*n* = 11)	
	Mean	SD	Mean	SD
*Assessment*				
Focused Thresholds (dB/1 μa)	43.0	6.8	45.6	4.2
Monopolar Thresholds (dB/1 μa)	32.0	4.4	32.7	4.4
Focused Threshold Variability (dB/1 μa)	2.5	0.9	2.6	1.0
Monopolar Threshold Variability (dB/1 μa)	1.7	1.1	1.9	1.0
Focused Dynamic Range (dB/1 μa)	10.2	5.6	10.6	3.7
Monopolar Dynamic Range (dB/1 μa)	11.5	3.7	11.8	3.5
Focused MCL (dB/1 μa)	52.4	5.2	55.7	3.9
Monopolar MCL (dB/1 μa)	42.9	2.7	44.0	2.7
Longitudinal Resistance (Ω)	644.00	895.0	515.00	544.0
Transverse Resistance (Ω)	22,178	17,509	15,242	8472
Total Resistance (Ω)	1433	696	1219	544
Clinical Electrode Impedance (Ω)	5509	2150	5562	2675

Means and standard deviations (SD) of values from each assessment for the child and adult groups, including auditory perceptual thresholds, channel-to-channel variability, dynamic range, MCLs, intracochlear resistance, and electrode impedance

**FIG. 2:**
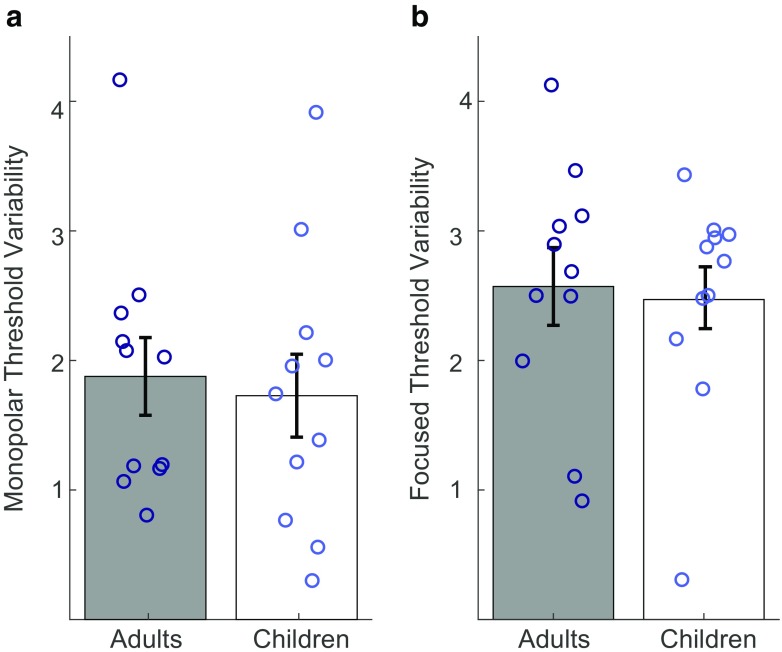
Channel-to-channel variability for late-implanted adults and early-implanted children. **a** Monopolar threshold variability. **b** Focused threshold variability. Bars represent average values for the adult group (in gray) and the child group (in white). Open circles overlaid on each bar depict individual data points. Adult values are dark blue and child values are light blue. Error bars represent one standard error above and below the mean

Channel-to-channel variability was defined as the standard deviation of the signed differences in threshold between neighboring electrodes. For the monopolar electrode configuration, channel-to-channel variability ranged from 0.29 to 3.91 dB in children and from 0.8 to 4.16 dB in adults (shown in Fig. 2). With the focused electrode configuration, threshold variability ranged from 0.3 to 3.43 dB in children and from 1.10 to 4.12 dB in adults. Average channel-to-channel variability was similar between groups. Larger average values were observed for focused compared with monopolar thresholds (means and standard deviations shown in Table 2). Statistical examination of threshold variance between early-implanted children and late-implanted adults revealed no significant effect of group (*F*(2,19) = 0.6, *p* = 0.943, Wilk’s ƛ = 0.994). Channel-to-channel variability did not differ significantly between children and adults for either electrode configuration (focused: *F*(1,20) = 0.07, *p* = 0.80; monopolar: *F*(1,20) = 0.10, *p* = 0.76).

**FIG. 3:**
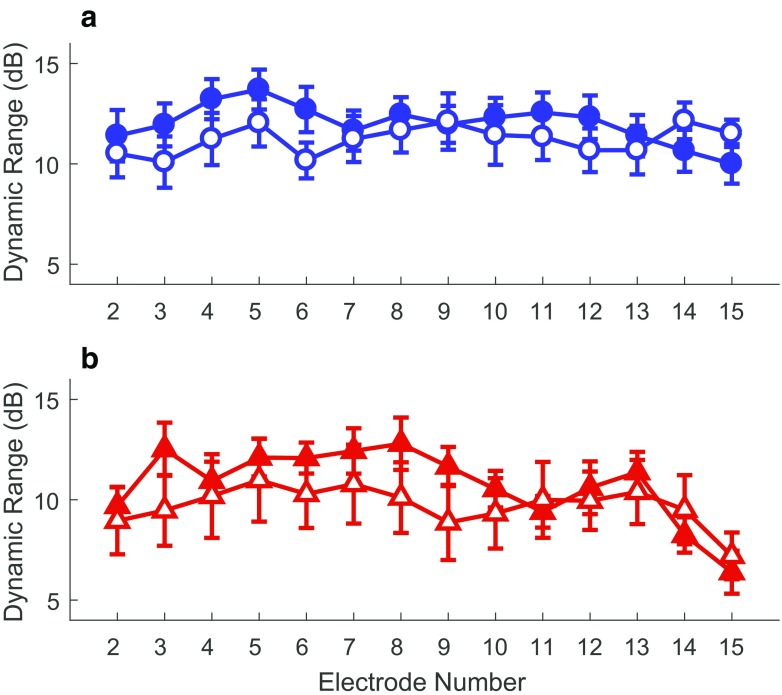
Dynamic range for late-implanted adults and early-implanted children across the electrode array. **a** Dynamic range from the monopolar electrode configuration. **b** Dynamic range from the focused electrode configuration. Adult data are filled symbols and child data are open symbols. Error bars represent one standard error above and below the mean

Similarly, as shown in Fig. 3, there was no significant effect of group on dynamic range, controlling for electrode site (*F*(2,259) = 0.30, *p* = 0.74, Wilk’s ƛ = 0.998). Average values were very similar between groups (see Table 2). The effect of electrode site on dynamic range was also not significant (*F*(2,249) = 3.9, *p* = 0.051, Wilk’s ƛ = 0.977).

**FIG. 4:**
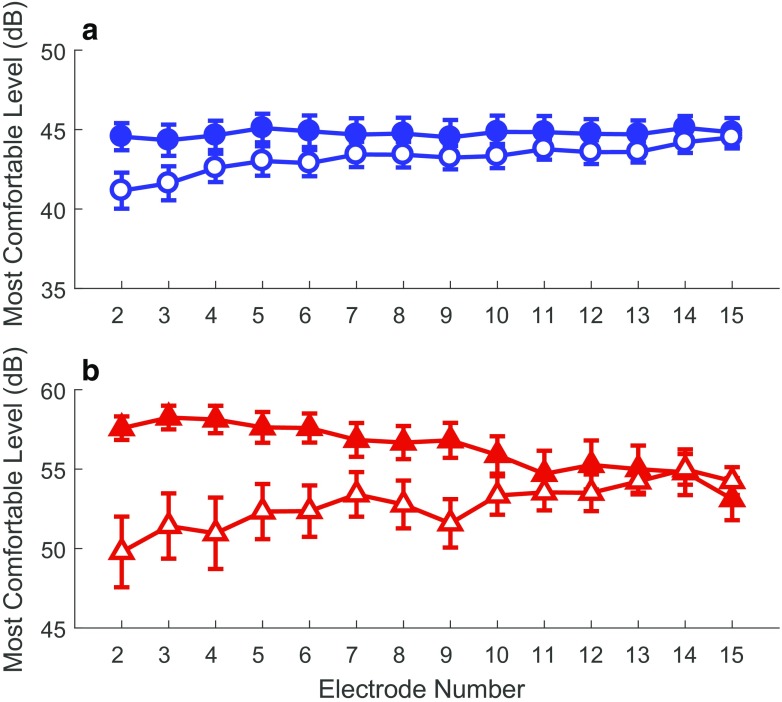
Most Comfortable Levels for late-implanted adults and early-implanted children across the electrode array. **a** Most Comfortable Levels from the monopolar electrode configuration. **b** Most comfortable levels from the focused electrode configuration. Adult data are indicated by filled symbols and child data by open symbols. Error bars represent one standard error above and below the mean

While dynamic range itself did not significantly differ between early-implanted children and late-implanted adults, a significant effect of group was found for the upper limit of the dynamic range (*F*(2,259) = 15.9, *p* < 0.001, Wilk’s ƛ = 0.891, partial *η*^2^ = 0.109). Children had significantly lower MCLs for the focused (*F*(1,260) = 31.9, *p* < 0.001, partial *η*^2^ = 0.1099) and monopolar (*F*(1,260) = 11.7, *p* = 0.001, partial *η*^2^ = 0.043) electrode configurations. Uncorrected means and standard deviations are in Table 2. The analysis also indicated a significant effect of electrode site on MCLs (*F*(2,259) = 6.7, *p* = 0.001, Wilk’s ƛ = 0.951, partial *η*^2^ = 0.049). This finding was significant for MCLs obtained with the monopolar configuration only (monopolar: *F*(1,260) = 9.2, *p* = 0.003, partial η^2^ = 0.034; focused (*F*(1,260) = 0.01, *p* = 0.93). MCLs obtained with monopolar stimulation demonstrated a consistent increase from the most apical (electrode 2) to the most basal (electrode 15) channel. This observation is akin to that of Baudhuin et al. 2012 who found higher MCLs for basal electrodes in children’s clinical programs. This effect in the present study was greater for the children than for the adults (see Fig. 4).

**FIG. 5:**
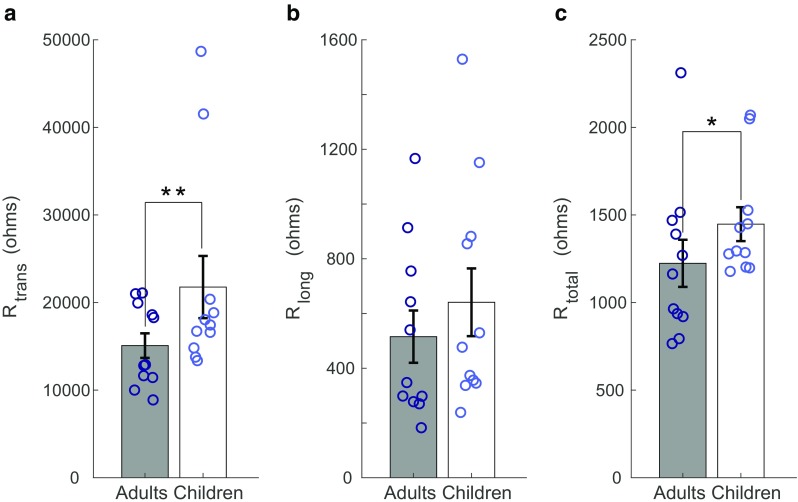
EFI intracochlear resistance values for late-implanted adults and early-implanted children. **a** Transverse resistance. **b** Longitudinal resistance. **c** Total resistance. Bars represent average values for the adult group (in gray) and the child group (in white). Open circles overlaid on each bar depict individual data points. Adult values are dark blue and child values are light blue. Error bars represent one standard error above and below the mean. *Difference is significant at *p* < 0.01. **Difference is significant at *p* < 0.001

Analysis of EFI intracochlear resistance values between early-implanted children and late-implanted adults revealed a significant effect of group (*F*(3,323) = 9.4, *p* < 0.001, Wilk’s ƛ = 0.920, partial *η*^2^ = 0.080) as well as electrode site (*F*(3,323) = 10.4, *p* < 0.001, Wilk’s ƛ = 0.912, partial *η*^2^ = 0.088) after correcting for multiple comparisons (ɑ = 0.017). Children demonstrated significantly higher R_trans_ (*F*(1,325) = 20.9, *p* < 0.001, partial *η*^2^ = 0.060) and R_total_ (*F*(1,325) = 9.9, *p* = 0.002, partial *η*^2^ = 0.030) compared with adults. No significant differences between groups were observed for R_long_ (*F*(1,325) = 2.7, *p* = 0.097), although on average, children did exhibit higher R_long_ values, as shown in Fig. 5. This non-significant result may be due to the large standard deviations in R_long_ values of children and adults (see Table 2). Electrode site was found to significantly affect R_total_ (*F*(1,325) = 6.6, *p* = 0.010, partial η^2^ = 0.020) and R_long_ (*F*(1,325) = 19.0, *p* < 0.001, partial η^2^ = 0.055). The location of the electrode in the array influenced the mean values for both groups: on average, both types of resistance were greater for basal electrodes compared with apical electrodes. Electrode site was not found to significantly influence R_trans_ (*F*(1,325) = 2.2, *p* = 0.135).

**Fig. 6:**
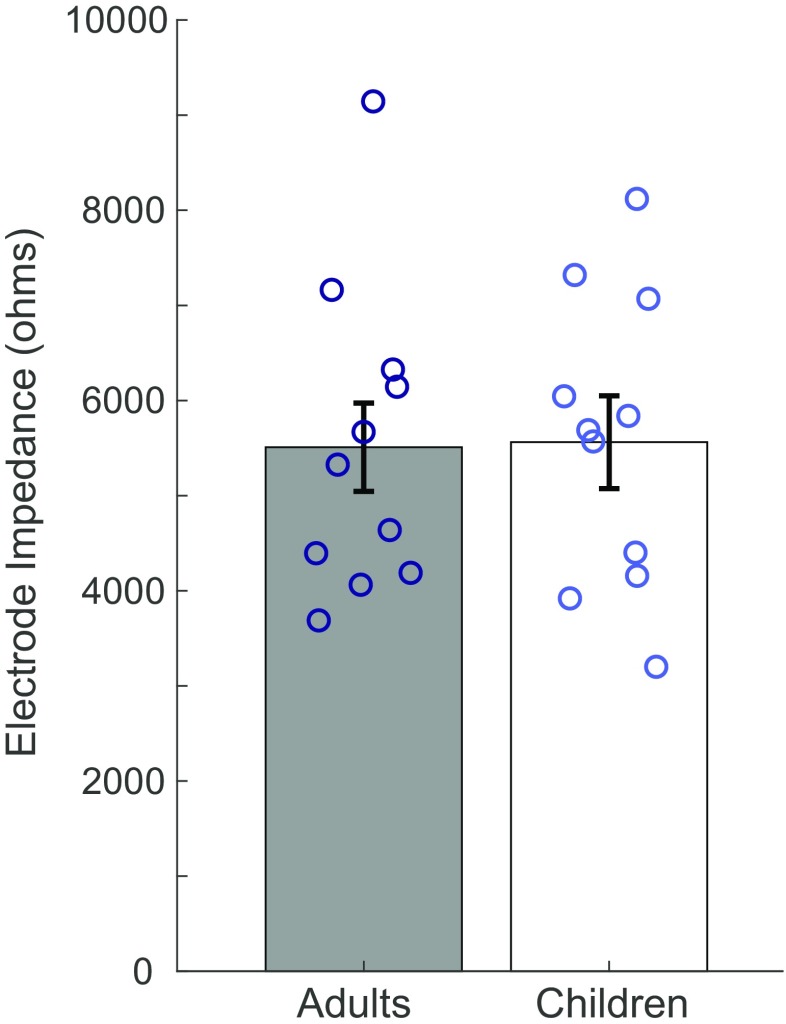
Clinical electrode impedance values for late-implanted adults and early-implanted children. Bars represent average values for the adult group (in gray) and the child group (in white). Open circles overlaid on each bar depict individual data points. Adult values are dark blue and child values are light blue. Error bars represent one standard error above and below the mean

Despite demonstrating significantly higher EFI component values, early-implanted children’s clinical electrode impedance values were similar to those of late-implanted adults (*F*(1,349) = 0.05, *p* = 0.82, partial *η*^2^ = 0; see Table 2 and Fig. 6). Electrode impedance was significantly affected by electrode location (*F*(1,349) = 74.7, *p* < 0.001, partial *η*^2^ = 0.176). Basal electrodes exhibited higher impedance levels than apical electrodes, similar to the results found for R_long_ and R_total_, and consistent with findings from previous studies in adults and children with CIs that controlled for physical characteristics of the electrode array (e.g., Leone et al. 2017; Molisz et al. 2015). EFI R_long_ values were found to significantly predict clinical electrode impedance along the electrode array (*F*(1,326) = 80.0, *p* < 0.001). This result suggests that these measures are related, and perhaps similarly affected by the degree of ossification and tissue growth in the cochlea.

## Discussion

This study compared factors related to the ENI in early-implanted children and late-implanted adults. It was hypothesized that the distinct demographics of these groups would differentially affect the ENI. In correspondence with this hypothesis, auditory detection thresholds for the focused electrode configuration were found to be significantly lower in early-implanted children compared with late-implanted adults. The difference between groups for monopolar thresholds was not significant, possibly because monopolar stimulation is less sensitive to ENI quality than is focused stimulation (Bierer 2007). It is unlikely that systematic between-group differences in electrode position contributed to this finding, as all participants had the same electrode array, and prior studies utilizing CT imaging have estimated similar percentages of poorly positioned electrodes between adult (Noble et al. 2014) and child (Noble et al. 2016) groups.

At present, a more probable explanation for children’s lower thresholds is that early-implanted children may have less neuronal degeneration, and consequently better ENI quality, compared with late-implanted adults. Individuals with severe hearing loss have lower spiral ganglion counts than their hearing counterparts (Otte et al. 1978). Evidence from cats indicates that spiral ganglion death occurs gradually in the absence of auditory stimulation (Leake and Hradek 1988). While the progression of spiral ganglion loss is believed to be slower in humans, these findings suggest that relatively long periods of deafness (which occurred for most adults in this study) reduce spiral ganglion survival. Normal aging has also been found to decrease spiral ganglion counts (Makary et al. 2011). However, electrical stimulation of spiral ganglion neurons in deafened animal models has been observed to promote survival of these neurons (Leake et al. 1991). Based on this evidence from animal models, it seems likely that early-implanted children would have greater neural survival (resulting from less neuronal degeneration) compared with late-implanted adults. In fact, neural responses obtained by Brown et al. (2010) suggested greater integrity of the auditory nerve in children with CIs compared with adults. The results of this investigation add further evidence to this premise.

Despite having lower auditory detection thresholds, pediatric CI users did not have larger dynamic ranges for either electrode configuration relative to adults because their MCLs were also significantly lower. The range of perceptible and comfortable current may be shifted to lower levels in early-implanted children. This finding is consistent with the idea that children have healthier and/or a greater density of spiral ganglion neurons, as lower levels of current in children elicited perceptual loudness ratings comparable to those of adults. Yet, MCLs of particular channels for only one child but those for six adult subjects could not be measured with focused stimulation without reaching the voltage compliance limits of the CI. This result suggests that a long duration or progression of hearing loss can influence the amount of current required to reach both threshold and MCL. However, this finding also indicates that the analogous values observed between groups for focused dynamic range should be interpreted with caution, as true MCL could not be assessed for many adults with that electrode configuration. The MCL and DR comparisons for focused stimulation are thus considered preliminary; further investigation with a larger sample of listeners who reach true MCL is required to confirm this result.

While the results described thus far suggest overall healthier ENIs in children, early-implanted children exhibited larger EFI intracochlear resistance compared with late-implanted adults. Previous research has demonstrated that intracochlear resistance values were positively related to the distance of electrodes from the inner wall of the cochlea (Bierer et al. 2015b). While both groups may have variation in electrode position within each of their CI electrode arrays, it is highly unlikely that all electrodes of pediatric CI users’ implants would be further away from the inner wall compared with adults, resulting in greater current resistance levels. This is particularly evident because children’s thresholds were found to be lower than those of the adult group: increased distance of the electrode from the target neurons would result in increased threshold for that electrode as well. Voltage is sensitive to the size of the electrode array and the cochlea (Vanpoucke et al. 2004), but the cochlea is adult-sized at birth (e.g., Pelliccia et al. 2014) and all participants in this study used the same type of electrode array without a positioner. Therefore, these factors should not have contributed to the EFI results.

The higher intracochlear resistances in early-implanted children may be due to greater levels of bone and tissue growth, which can increase resistance to electrical current flow (Spelman et al. 1982). Ossification and fibrous tissue growth have been found to occur after cochlear implantation in adults due to trauma from electrode array insertion (Li et al. 2007; Seyyedi and Nadol 2014). Children could have greater cochlear ossification and tissue growth within the cochlea after CI surgery compared with adults because temporal bone growth is dynamic throughout childhood (Dahm et al. 1993).

However, clinical electrode impedance levels (which have been found to increase with tissue growth around the electrodes; e.g., Wilk et al. 2016) were comparable between early-implanted children and late-implanted adults in the present study. The R_long_ EFI component was found to significantly relate to electrode impedance, but these are not the same measure: EFI characterizes the resistivity of the tissue around the electrodes instead of the electrodes themselves and provides information about the physical characteristics of the fluid-filled space surrounding the electrode array. In addition, electrode impedances are dominated by the capacitance across the metal-tissue interface whereas EFI-derived impedances are not (Hanekom 2005). It is possible that children have similar tissue growth around the electrode array, but increased ossification and tissue formation in the full cochlear space, relative to adults.

While the finding of increased intracochlear resistance in children may seem contradictory to the lower auditory perceptual thresholds observed in this group, these results may be consistent with Ohm’s Law (Voltage = Impedance*Resistance). Increased intracochlear resistance (R) in children would lead to less current flow (I) along the cochlea and away from the neural targets to equal the same driving voltage gradient (V) at the spiral ganglion as in the late-implanted adults. Less current (I) would therefore be required to achieve threshold in individuals with higher intracochlear resistance (R). Continued study is needed to better understand how the complex geometry of the cochlea affects the spread of excitation with CI stimulation.

An exploratory regression analysis was performed to investigate whether intracochlear resistance levels of the participants in this study affected auditory detection thresholds. A linear mixed-effects model was performed in R using the lme4 package (Bates et al. 2015). Thresholds across the electrode array were specified as the dependent variable, with group membership, measures of intracochlear resistance, and type of electrode configuration for obtaining thresholds as the independent variables. “Subject” was included as a random intercept in the model. Results indicated a significant relation of R_long_, which reflects global current flow within the cochlea, to thresholds of participants (*β* = 0.49, standard error = 0.14, *p* < 0.001). A significant interaction between “Group” and “Stimulus Type” was also found (*β* = 1.9, standard error = 0.48, *p* < 0.001), demonstrating that thresholds were significantly different between groups even when intracochlear resistance was taken into account. Altogether, these results indicate that hearing demographics can alter physical characteristics of the cochlea that influence auditory signal transmission.

Consistent with previous studies in adults (e.g., Bierer 2007; Bierer et al. 2015a; Long et al. 2014), both pediatric and adult CI users in this study exhibited relatively uniform monopolar thresholds, but variable focused thresholds, across the electrode array. No significant differences in channel-to-channel variability were observed between groups. It is important to note that a prior study has observed asymmetric levels of stimulation between sQP channels: Padilla et al. (2017) found nonmonotonic increases in the center of gravity of sQP stimuli that were associated with alterations in place pitch perception. It is unclear how this issue would affect perceptual thresholds, and there is no indication that the magnitude of this effect would differ between the children and adults in the present study. However, asymmetric current steering may limit the ability to detect true channel-to-channel threshold variability. It is thus possible that this contributed, in part, to the finding that threshold variability did not differ between groups.

Examinations of individual participant data revealed that children had regions of relatively elevated focused thresholds within their threshold profiles, akin to late-implanted adults. Although early implantation has been associated with better outcomes for children (e.g., Wang et al. 2008), this result suggests that even early-implanted children have electrode-neuron interfaces that are of poorer quality than others within their implant. Several prior studies have shown that experimental programming processing strategies or stimulation types can improve speech perception in CI users (e.g., Arenberg et al. 2018; Garadat et al. 2013). However, all but one of such studies (e.g., Noble et al. 2016) have been conducted in adults (Azadpour and Smith 2016; Koning and Wouters 2016; Müller et al. 2018; Nogueira et al. 2016; Padilla et al. 2017), most of whom were postlingually deafened and late-implanted. Techniques which have been used in adults to improve transmission of auditory signals through suboptimal ENIs could be applied in children (e.g., Bierer and Litvak 2016).

Further, although the relation between spectral discrimination and speech identification scores in pediatric CI users may depend on the tests utilized (e.g., DiNino & Arenberg, 2018; Gifford et al. 2018), current interaction between channels could be more problematic for children if they do indeed have greater neuronal densities (Jahn et al. 2018). Children could potentially benefit from processing strategies that use focused electrode configurations to reduce this channel interaction, which have had only minimal success in some adults (Berenstein et al. 2008; Srinivasan et al. 2013). Future studies on optimization of programming strategies for early-implanted children are warranted.

In conclusion, the differences related to the ENI between early-implanted children and late-implanted adults may be explained by neuronal survival, etiology of deafness, or other factors related to intracochlear characteristics. Future studies should investigate the physiological mechanisms that contribute to ENI quality in these groups. Investigations similar to the present study could also be extended to other CI user populations, such as children who had progressive hearing loss or adults who lost their hearing as children. Such studies could provide valuable information about the effect of hearing demographics on auditory signal transmission and perception of individuals with CIs.
